# Dynamics of the vibro-impact capsule robot with a *von Mises* truss

**DOI:** 10.1007/s11071-024-10653-4

**Published:** 2024-12-08

**Authors:** Yao Yan, Joseph Páez Chávez, Jiajia Shen, Yang Liu

**Affiliations:** 1 School of Aeronautics and Astronautics, University of Electronic Science and Technology of China, Chengdu, 611731 China; 2 Center for Applied Dynamical Systems and Computational Methods (CADSCOM), Faculty of Natural Sciences and Mathematics, Escuela Superior Politécnica del Litoral, P.O. Box 09-01-5863, Guayaquil, Ecuador; 3 Department of Mathematics, Center for Dynamics, TU Dresden, 01062 Dresden, Germany; 4 Exeter Technologies Group, Department of Engineering, University of Exeter, North Park Road, Exeter, EX4 4QF UK; 5 Exeter Small-Scale Robotics Laboratory, Engineering Department, University of Exeter, North Park Road, Exeter, EX4 4QF UK

**Keywords:** Vibro-impact, Capsule robot, *von Mises* truss, Functionalised nonlinear structures, Bifurcation analysis, Numerical continuation

## Abstract

Functionalised nonlinear structures employing structural instabilities for rapid response shape-shifting are emerging technologies with a wide range of potential applications. The *von Mises* truss is a widely employed model for such functionalised nonlinear structures; however, few studies have delved into its functionality when integrated with a complex dynamical system. This paper investigates its efficacy on enhancing the progression speed of a vibration-driven robot, known as the vibro-impact capsule robot, which is a piecewise-smooth dynamical system having abundant coexisting attractors. Bifurcation analysis of the capsule robot integrated with a *von Mises* truss is conducted for this purpose. Our numerical studies focus on the influence of the frequency and amplitude of the robot’s driving force on its progression. Specifically, we compare the periodic responses of both the capsule robots with and without the *von Mises* truss, utilising the numerical continuation techniques for piecewise-smooth dynamical systems. Our studies confirm the advantage of using the *von Mises* truss when the driving force of the robot is significantly small. Additionally, we identify an optimal operational regime on the amplitude-frequency control plane, where the maximum robot speed is achieved for a given amount of power consumption. The numerical studies presented in this work provide a promising indication of the advantages offered by the *von Mises* truss for vibration-driven robots. This research underscores the significant potential of functionalised nonlinear structures to enhance the efficiency of small-scale robots operating under power limitations.

## Introduction

Scientific progress in the design and control of robots presents exciting opportunities across diverse engineering sectors, e.g., healthcare [1] and energy [2]. Nevertheless, robotic engineers encounter significant challenges in ensuring the resilience and competitiveness of robots within their environments. Consequently, control performance often suffers due to inherent or external nonlinearities arising from mechanical structures or environmental interference, respectively. Within this realm, small-scale robots [3], ranging from millimeters to micrometers, confront unique obstacles. These diminutive robots find applications in areas such as minimally invasive surgical tools in clinical medicine [4–6] and aerial robots for environmental exploration and manipulation [7]. However, the limited range of structures and materials available for these robots poses difficulties in achieving desired performance and operational modes. The constrained force or torque outputs of miniature actuators, as well as power limitations, further restrict their performances. Therefore, the development of innovative engineering solutions for small-scale robots to enhance their robustness and efficiencies becomes imperative.

In the present work, we aim to study the performance enhancement for a vibration-driven robot, known as the vibro-impact capsule robot [8, 9], by employing the structural instability of a *von Mises* truss [10]. The operation of a vibro-impact capsule robot relies on the rhythmic excitation of an internal mass, such as a magnet driven by an external magnetic field, within a capsule-like structure. This mass functions analogous to a “hammer” engaging with the primary body of the robot to induce movement in the presence of external resistances [11]. Similarly, a micromachined actuator adopting the same driving principle was developed by Mita et al. [12]. However, its progression performance is extremely low. Also, such a robot consists of several nonlinearities that are experienced by many small-scale robots, such as oscillations [13], frictions [14] and collisions [15]. From a nonlinear dynamics point of view, the vibro-impact capsule robot is a piecewise-smooth dynamical system [16] which has abundant coexisting attractors [17]. This implies that multiple modes of operation may coexist in the same system configuration. Among these attractors, only those associated with specific motion patterns, e.g., a period-1 forward motion, are of interest for control purposes. Consequently, understanding its nonlinearity that involves addressing several key research issues, such as the emergence and switching, annihilation of multiple undesired attractors, was the focus of past research, see e.g., [18, 19]. Such behaviours are typical in small-scale robots, which thus provides a rationale for using this robot as a benchmark system in the present work.

Structural instabilities, which were treated as failure modes [20–22], have been increasingly exploited for functionality, known as *buckliphilia* [23] or *well-behaved nonlinear structures* [24]. Their applications include (but not limited to) programmable mechanical metamaterial [25–27], fast response shape shifting structures [28–31], energy harvesters [32, 33], pattern formation for the manufacturing of flexible electronics [34–36], as well as non-destructive testing techniques [37–39]. While previous studies have focused on the nonlinear response of individual structures or materials, a comprehensive understanding of their nonlinear behaviour has proven sufficient for ensuring functionality. However, few studies have investigated the integration of these nonlinear structures into complex dynamical systems. This paper addresses the gap by exploring how the functionality of a nonlinear structure is achieved through its interaction with the overall system.

One of the key challenges in the design of *well-behaved nonlinear structures* is the robust analysis of their nonlinear behaviour. Exploring the entire design space through nonlinear analysis can be computationally expensive, and existing commercial finite element packages lack facilities for strict bifurcation analysis, such as pinpointing critical points, switching branches at bifurcation points, and tracing specific equilibrium points related to certain bifurcation points [24]. This limitation confines existing well-behaved structures to a limited number of well-understood structural forms [30]. On the other hand, classical simple toy models, composed of rigid bars and springs and solvable analytically, continue to play a crucial role in unraveling the intricate mechanics of well-behaved nonlinear structures and in the development of novel applications [40–43]. The *von Mises* truss is one of the most widely used models of this type. By tailoring the geometry and the properties of its component springs, a diverse array of nonlinear behaviours can be obtained [44, 45]. Considering its simplicity and rich representative nonlinear behaviour, we adopt the *von Mises* truss as the nonlinear structural spring to replace the existing linear counterpart in this paper.

In contrast to traditional robotic systems, capsule robots, e.g., [46–48], exhibit the ability to access narrower and more delicate areas, such as the gastrointestinal tract, while minimising potential damage. Nevertheless, their compact dimensions pose challenges in integrating essential components (e.g., actuators, controllers and sensors) on-board. The current reliance on externally magnetic control solutions may require additional space and facilities for manipulation. In particular, during the prolonged operations, patients may be exposed to a strong magnetic field causing other side effects. Thus, enhancing the capability of on-board driving is crucial for the manipulation of small-scale robots. In [9], the driving force generated from an external coil was up to 200 mN, but as the distance between the coil and the capsule robot increased to 60  mm such a driving force dropped to 50 mN. Similar results were obtained by Zhang et al. [6] where a hand-held coil was used to drive a colon capsule robot. The coil needs to be held at a distance of 40  mm away from the robot to ensure a sufficient driving force acting on the robot. A novel on-board actuator was developed by Zhang et al. [46], but its driving force was only up to 10 mN. Therefore, in this work, we will explore how to utilise the structural instability of a *von Mises* truss to magnify the driving force of the capsule robot. Specifically, we will show how to boost the average speed of the robot through carefully selecting its control parameters (i.e., frequency and amplitude of robot’s driving force).

In this work, we will perform a numerical study of the harmonically excited capsule robot equipped with a nonlinear *von Mises* truss as described above, using numerical continuation (path-following) techniques for piecewise-smooth dynamical systems. Specifically, we will employ the numerical continuation platform COCO [49] (a MATLAB-based analysis and development platform for the numerical solution of continuation problems) to perform a bifurcation analysis of the periodic response of the capsule robot. For this purpose, we will extensively utilise the COCO-toolbox ‘hspo’, which extends and improves the functionalities of the software package TCHAT [50] (an AUTO-based application for continuation and bifurcation detection of periodic orbits of piecewise-smooth dynamical systems). Both platforms divide a periodic solution into smooth segments characterised by a smooth vector field representing the system’s behaviour within the segment and an event function defining the segment’s terminal point. This mathematical framework will be introduced in detail in this study. In addition, our numerical investigation will include physical quantities such as average capsule velocity and power consumption measured for a certain periodic response. By utilising the continuation capabilities of COCO, we aim to identify a locus on the amplitude-frequency control plane that achieves constant power consumption levels while maintaining varying capsule velocity. This will enable us to determine an optimal operational regime characterised by maximum capsule speed.

**Fig. 1:**
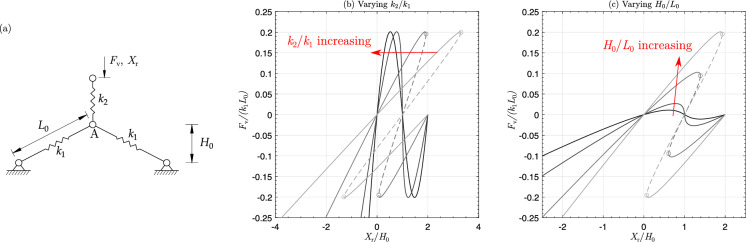
**a** Geometry and stiffness layout of the *von Mises truss*. **b** Equilibrium paths of *von Mises truss* with $k_2/k_1=0.1,~0.2,~1,~10$ and $H_0/L_0=0.72$. **c** Equilibrium paths of *von Mises truss* with $H_0/L_0=0.3,~0.4,~0.6,~0.72$ and $k_2/k_1=0.2$. The solid and dashed curves represent the stable and unstable equilibria under displacement-controlled loading. The hollow circles represent the limit points where snap-down occurs

The rest of the paper was organised as follows. In Sect. 2, mathematical models of the *von Mises* truss and its integration with the capsule robot were studied. In Sect. 3, bifurcation analysis of the capsule robot with the *von Mises* truss was carried out through Rung-Kutta simulations. Our studies focused on the influence of frequency and amplitude of robot’s external excitation on the average velocity of the robot. Then we compared the periodic responses of both the capsule robots with and without the *von Mises* truss by using path-following techniques in Sect. 4. Finally, conclusions are drawn in Sect. 5.

## Mathematical models

### *von Mises* truss

Figure 1a shows a *von Mises* truss connected in series with linear springs. The structure has an arch-like arrangement of two inclined linear springs $k_1$, with a third linear spring $k_2$ suspended above the apex [45]. The structure is subjected to a load/displacement actuation at the top end of the vertical spring $k_2$. The reaction force–displacement ($F_\textrm{v}$ versus $X_\textrm{r}$) response at the actuation point is a sigmoidal curve. The characteristics of this sigmoidal equilibrium path are defined by the geometric arrangement (the span $L_0$ and the rise $H_0$) and the stiffness ratio of the inclined versus vertical springs ($k_2/k_1$). The equilibrium path is defined by the governing equation:

1 $$\begin{aligned} \begin{aligned} F_\textrm{v} = \frac{2 \, k_{1} \, \left( L_0 - \sqrt{L_0^{2} - H_{0}^{2} + \left( H_{0} - u_\textrm{apx}\right) ^{2}}\right) \, \left( H_{0} - u_\textrm{apx}\right) }{\sqrt{L_0^{2} - H_{0}^{2} + \left( H_{0} - u_\textrm{apx}\right) ^{2}}} \end{aligned} \end{aligned}$$

where $u_\textrm{apx}$ is the vertical displacement at the apex of the *von Mises* truss, i.e. Point A in Fig. 1a. The vertical displacement at the actuation point $X_\textrm{r}$ can be written as:

2 $$\begin{aligned} X_\textrm{r}=\frac{F_\textrm{v}}{k_2}+u_\textrm{apex}. \end{aligned}$$

Figure 1b, c shows the equilibrium paths with selected $k_2/k_1$ and $L_0/H_0$ ratios. With the increase of $k_2/k_1$, the snap-backs in the equilibrium path disappear and the system becomes monotonically stable under the displacement-controlled loading. The initial stiffness of the system increases while the peak load remains the same. With the increase of $H_0/L_0$, the initial stiffness and the peak load of the system increases. The system can exhibit snap-backs in the nonlinear equilibrium path.

**Fig. 2:**
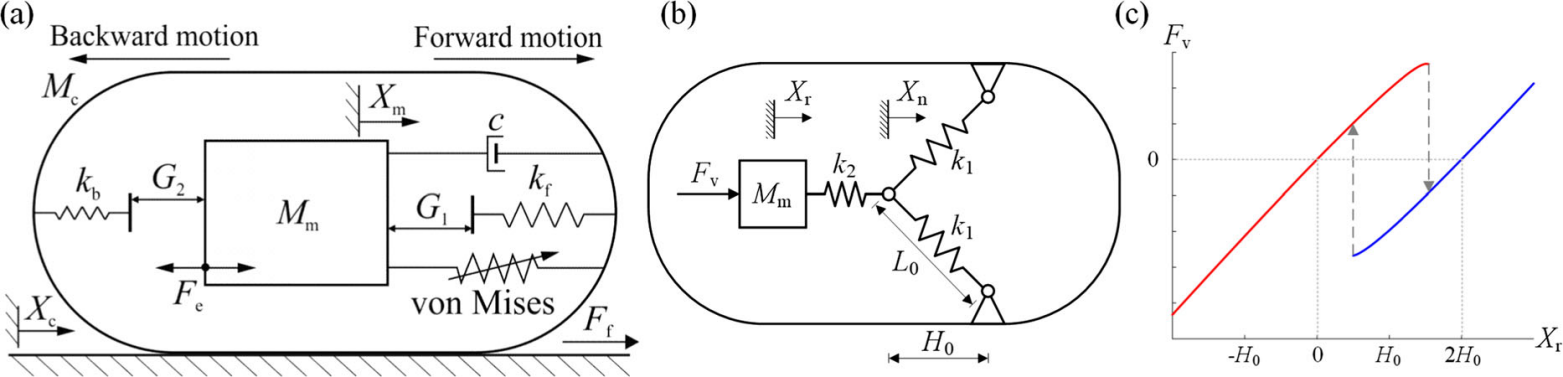
Components of the vibro-impact capsule system. **a** Physical model of the vibro-impact capsule system. **b** Schematic representation of the *von Mises* truss, highlighting the primary connection between the inner mass and the capsule shell. **c** Nonlinear reaction force on the *von Mises* truss as a function of the relative displacement between the inner mass and the capsule shell

Due to the inherent symmetry of geometry, the equilibrium path of the *von Mises* truss is anti-symmetric about the equilibrium state characterised by $X_\textrm{r}/H_0=1$ and $F_\textrm{v}=0$. This state corresponds to the configuration when the two inclined springs $k_2$ are aligned horizontally. If we load the *von Mises* truss from the initial state with $F_\textrm{v}=0$ and $X_\textrm{r}=0$, the system exhibits asymmetric behaviour under compression and tension, particularly when the compression displacement surpasses the critical point, leading to negative stiffness. Therefore, to make the system work differently from the linear counterpart, we should ensure that the effective compression displacement on the nonlinear spring surpasses the force limit point, resulting in negative stiffness.

### Capsule robot integrated with *von Mises* truss

The considered system, depicted in Fig. 2a, operates in bidirectional stick–slip phases encompassing four distinct modes: *stationary capsule without impact*, *moving capsule without impact*, *stationary capsule with impact* and *moving capsule with impact*. All these modes can be modelled via the following equations of motion

3 $$\begin{aligned} \left\{ \begin{array}{lll} M_\textrm{m}\ddot{X}_\textrm{m}& =& F_\textrm{e}-F_\textrm{i},\\ M_\textrm{c}\ddot{X}_\textrm{c}& =& F_\textrm{f}+F_\textrm{i}, \end{array} \right. \end{aligned}$$

where $F_\textrm{e}$ is the external excitation, $F_\textrm{f}$ is the friction acting on the capsule, and $F_\textrm{i}$ represents the interaction force between the capsule and the magnet written as

4 $$\begin{aligned} F_\textrm{i}=\left\{ \begin{array}{ll} F_\text {v}+cV_\textrm{r}+F_2, & \quad X_\textrm{r} \le -G_2,\\ F_\text {v}+cV_\textrm{r}, & \quad -G_2 \le X_\textrm{r} \le G_1,\\ F_\text {v}+cV_\textrm{r}+F_1, & \quad X_\textrm{r} \ge G_1. \end{array} \right. \end{aligned}$$

Here, $X_\textrm{r}=X_\textrm{m}-X_\textrm{c}$ and $V_\textrm{r}=V_\textrm{m}-V_\textrm{c}$ represent the relative displacement and velocity between the magnet and the capsule. The interaction forces for the front and back constraints are represented by $F_1=k_f (X_\textrm{r} - G_1)$ and $F_2=k_b (X_\textrm{r} + G_2)$, respectively. The *von Mises* truss, as shown in Fig. 2b, can be written as

5 $$\begin{aligned} {\left\{ \begin{array}{ll} F_\text {v}=k_2 \left( X_\text {r} - X_\text {n} \right) ,\\ F_\text {v}=2k_1\left( L_0-\sqrt{L_0^2-H_0^2 +\left( H_0-X_\text {n}\right) ^2}\right) \\ \qquad \qquad \frac{H_0-X_\text {n}}{\sqrt{L_0^2-H_0^2 +\left( H_0-X_\text {n}\right) ^2}}. \end{array}\right. } \end{aligned}$$

For any given relative displacement, $X_\text {r}$, the displacement of the node point and the force of the *von Mises* truss, $X_\text {n}$ and $F_\text {v}$, can be obtained by numerically solving Eq. (5). Given the nature of multiple solutions of the hysteresis (either continuous change or sudden jump), Newton iteration is adopted with the initial guess chosen as the latest solution. In general, the *von Mises* truss is nonlinear, except the special cases where $k_1$ is extremely large. For some parameter combinations, the *von Mises* truss displays a hysteresis loop, as seen in Fig. 2c. Namely, there is a region in the vicinity of $X_\text {r}=H_0$ where $F_\text {v}$ has two values for a given relative displacement.

**Table 1:** System and control parameters of the capsule robot with the *von Mises* truss

Parameters	Unit	Values
$M_\textrm{m}$	$\textrm{g}$	1.8
$M_\textrm{c}$	$\textrm{g}$	1.67
$\mu$	−	0.59
$G_1$	$\textrm{mm}$	0.8
$G_2$	$\textrm{mm}$	0.8
$k_1$	$\mathrm {kN/m}$	0.4
$k_2$	$\mathrm {kN/m}$	0.062
*c*	$\mathrm {Ns/m}$	0.0156
$H_0$	$\textrm{mm}$	0.4
$L_0$	$\textrm{mm}$	1.0
$k_\textrm{f}$	$\mathrm {kN/m}$	53.5
$k_\textrm{b}$	$\mathrm {kN/m}$	27.9
$P_\textrm{d}$	$\textrm{mN}$	[10, 60]
$\Omega$	$\textrm{Hz}$	[5, 40]

Physical parameters of the robot were adopted from our previously reported prototype [9]. Physical parameters of the *von Mises* truss were reasonably selected based on the dimension of our robot prototype and the feasibility of fabricating such a truss in real-world scenarios

In this study, the frictional force between the capsule and the supporting surface is given as

6 $$\begin{aligned} \left\{ \begin{array}{lrl} F_\textrm{f}\in [-P_\textrm{f},\,P_\textrm{f}],&  & V_\textrm{c}=0,\\ F_\textrm{f}=-\textrm{sign}(V_\textrm{c})P_\textrm{f},&  & V_\textrm{c}\ne 0, \end{array} \right. \end{aligned}$$

where $P_\textrm{f}=\mu (M_\textrm{m}+M_\textrm{c})g$ is the static friction of the capsule, and *g* is the gravitational acceleration. The external excitation, $F_\textrm{e}$, is a sinusoidal excitation written as

7 $$\begin{aligned} F_\textrm{e}(t)=P_\textrm{d}\sin (2\pi \Omega t), \end{aligned}$$

where $P_\textrm{d}$ and $\Omega$ are the amplitude and frequency of the excitation, respectively. Note that the analytical formulation presented here has been validated using nonlinear finite element modeling and simulation in the commercial software Abaqus. Excellent comparison between the finite element and analytical simulation is observed. However, compared with finite element simulation, the analytical method is much more computationally efficient. Detailed information can be found in our published work [51].

System and control parameters of the capsule robot and the physical parameters of the *von Mises* truss are summarised in Table 1.

## Bifurcation analysis

Next, we conducted analysis based on the governing equations of motion in Eq. (3) to study the progression performance of the capsule with a *von Mises* truss, as depicted in Fig. 2. This was realised by employing fourth-order Rung-Kutta simulations, with the time step set as four thousandth of a driven period and 60 periods calculated. Moreover, the first 40 driven periods were omitted to skip the transient phase, and the maximum relative displacement, $X_\textrm{r}^*$, achieved in each excitation period was recorded and plotted as functions of the driven frequency. Then the displacements in the last 20 periods were used to calculate the average progression speed, $V_\text {ave}$, by dividing them using the total elapsed time. Given the nonlinearity of this capsule robot, brute-force simulations were carried out by increasing and decreasing bifurcation parameters to search potential co-existing attractors. Namely, the capsule’s final state in the last simulation, including the relative displacement, velocities of the capsule and the inner mass, was used as the initial condition of next simulation.

**Fig. 3:**
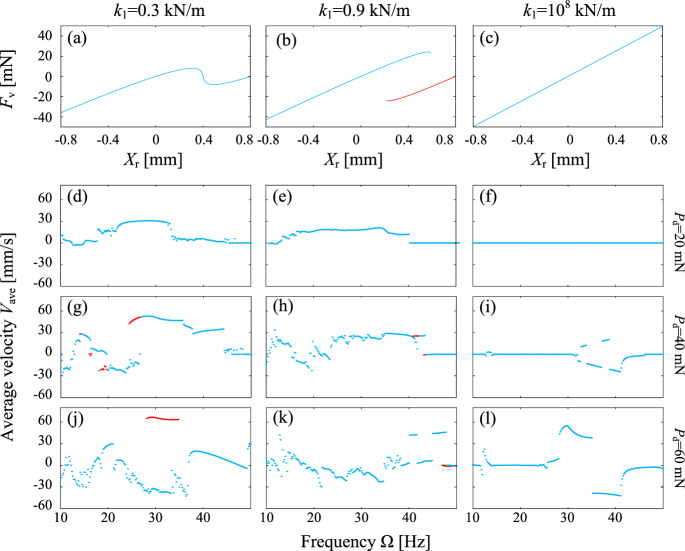
Reaction force of the *von Mises* truss, $F_v$, as a function of the relative displacement, $X_\textrm{r}$, for **a** $k_1=0.3$ kN/m, **b** 0.9 kN/m and **c** $10^8$ kN/m, where the red line indicates a discontinuous branch in case of hysteresis. The spring displays properties of negative stiffness, hysteresis, and linear stiffness, respectively. With each column corresponding to the stiffness, the average progression speed of the capsule varies with respect to the change of driven frequency, where the driven force is fixed as **d**–**f** $P_\textrm{d}=20$ mN, **g**–**i** 40 mN, and **j**–**l** 60 mN, respectively, where the red dots indicate co-existing attractors in case of bistability

As displayed in Fig. 3a, the *von Mises* spring has a negative stiffness in the vicinity of $X_\text {r}=H_0=0.4$ mm, when the two inclined springs have a relatively small stiffness, $k_1=0.3$ kN/m. For a relatively large stiffness of the inclined springs in Fig. 3b, $k_1=0.9$ kN/m, the *von Mises* truss displays a hysteresis loop in the vicinity of $X_\text {r}=H_0$. For an exceptionally large stiffness of $k_1=10^8$ kN/m, as represented in Fig. 3c, the inclined spring hardly deform and the *von Mises* truss degenerates into a linear spring with a stiffness of approximately $k_2$. In this scenario, the capsule introduced in Fig. 2 becomes the classical vibro-impact capsule discussed in our previous studies [6, 8].

Note that we have conducted an extensive parametric study on the effects of negative stiffness and snap-back on the overall propelling performance of the capsule robot in authors’ recent work [51]. We have identified that the negative stiffness of the nonlinear structural spring will lead to a wider frequency region where the capsule remain high velocity, i.e. their insensitivity to the excitation frequency variation. However, in this work, we mainly focus on the detailed dynamic response of the system and how they affect the velocity of the capsule robot.

Subsequently, we used the driven frequency, $\Omega$, as the control parameter, with all of the other parameters fixed to study the progression velocities of the capsule. Results are displayed in Fig. 3d–l, where the left, middle and right columns have $k_1=0.3$ kN/m, 0.9 kN/m and $10^8$ kN/m, and the first, second and third rows have $P_{\textrm{d}}=20$ mN, 40 mN and 60 mN, respectively. It is seen that the *von Mises* truss with negative stiffness always yields the largest progression speed in Fig. 3d, g and j compared with the other cases. For a small driven force of $P_{\textrm{d}}=20$ mN, Fig. 3f shows that the classical vibro-impact capsule does not progress at all. However, this excitation amplitude is sufficient for the capsules with $k_1=0.3$ kN/m and 0.9 kN/m to move forward. The corresponding progression speed in Fig. 3d–e is especially evident for $\Omega \approx 30$ Hz.

The progression speed for $P_\textrm{d}=40$ mN is represented in Fig. 3g–i, where Fig. 3i shows that the classical vibro-impact capsule progress for $\Omega >30$ Hz, and its moving direction alters as the driven frequency increases. For $k_1=0.9$ kN/m, the hysteresis loop introduces significant complexity in the progression speed variation depicted in Fig. 3h compared with that in Fig. 3e for a small driven force. In particular, the progression speed exhibits discontinuities with increasing driven frequency. By contrast, Fig. 3g shows that the spring with negative stiffness consistently yields the highest progression speed, reaching $V_\text {ave}=52.77$ mm/s for $\Omega =28.6$ Hz. However, for $\Omega \in [24.4, 26.6]$ Hz, there exists a coexistence between states with fast forward ($V_\text {ave}\ge 42.5$ mm/s) and slow backward motions.

A further increase of the driven force to $P_\textrm{d}=60$ mN noticeably enhances the performance of the classical vibro-impact capsule with a linear spring. The peak progression speed in Fig. 3l reaches $V_\text {ave}=55.03$ mm/s, attained at $\Omega =29.8$ Hz. Simultaneously, the largest driven force makes the progression speed and direction in Fig. 3k for the *von Mises* truss with hysteresis loop even more unpredictable. The complex trend indicates that the hysteresis introduces profound bifurcations in capsule’s response with respect to the driven frequency. For $k_1=0.3$ kN/m, the negative stiffness with large driven force also lead to a complex change of the progression speed in Fig. 3j, particularly for $\Omega <40$ Hz. Nevertheless, Fig. 3j presents a region, $\Omega \in [28.2, 34.8]$ Hz, where high-speed forward capsule progression coexists, achieving the maximum speed of $V_\text {ave}=66.92$ mm/s at $\Omega =29.2$ Hz.

**Fig. 4:**
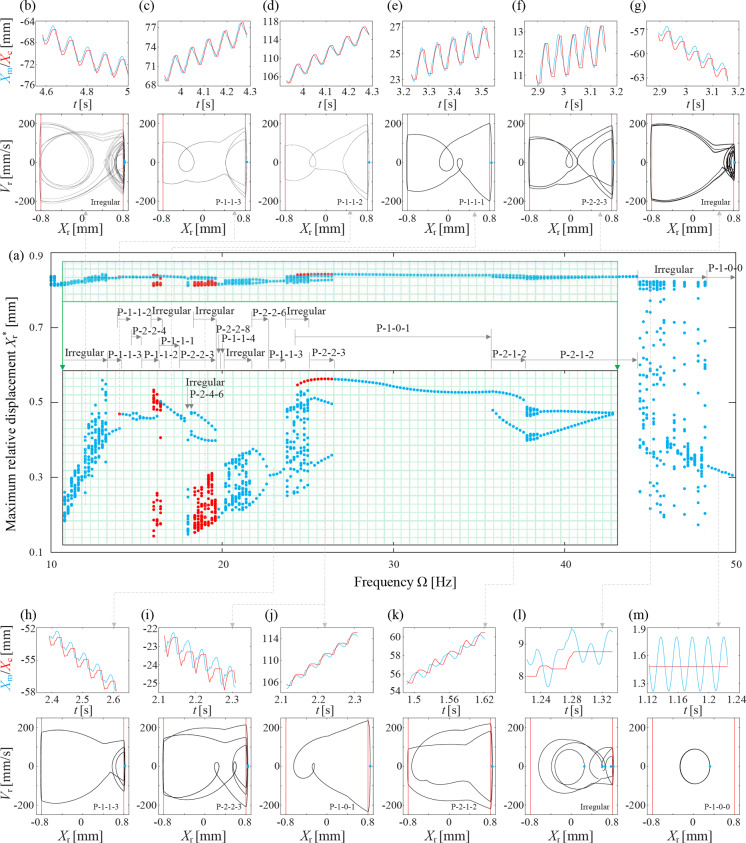
**a** Bifurcation diagram of the capsule response for $k_1=0.3$ kN/m, where the maximum relative displacement, $X_\textrm{r}$, is displayed as a function of the driven frequency. In addition, the region for $\Omega \in [10.8, 41]$ Hz has been enlarged to display the details within the green area. Corresponding time series and phase portraits for **b** $\Omega =12$ Hz, **c**, **d** 14 Hz, **e** 17 Hz, **f**,**g** 19 Hz, **h** 23 Hz, **i**, **j** 26 Hz, **k** 37 Hz, **l** 45 Hz, and **m** 49 Hz, have been added to show different capsule responses

**Fig. 5:**
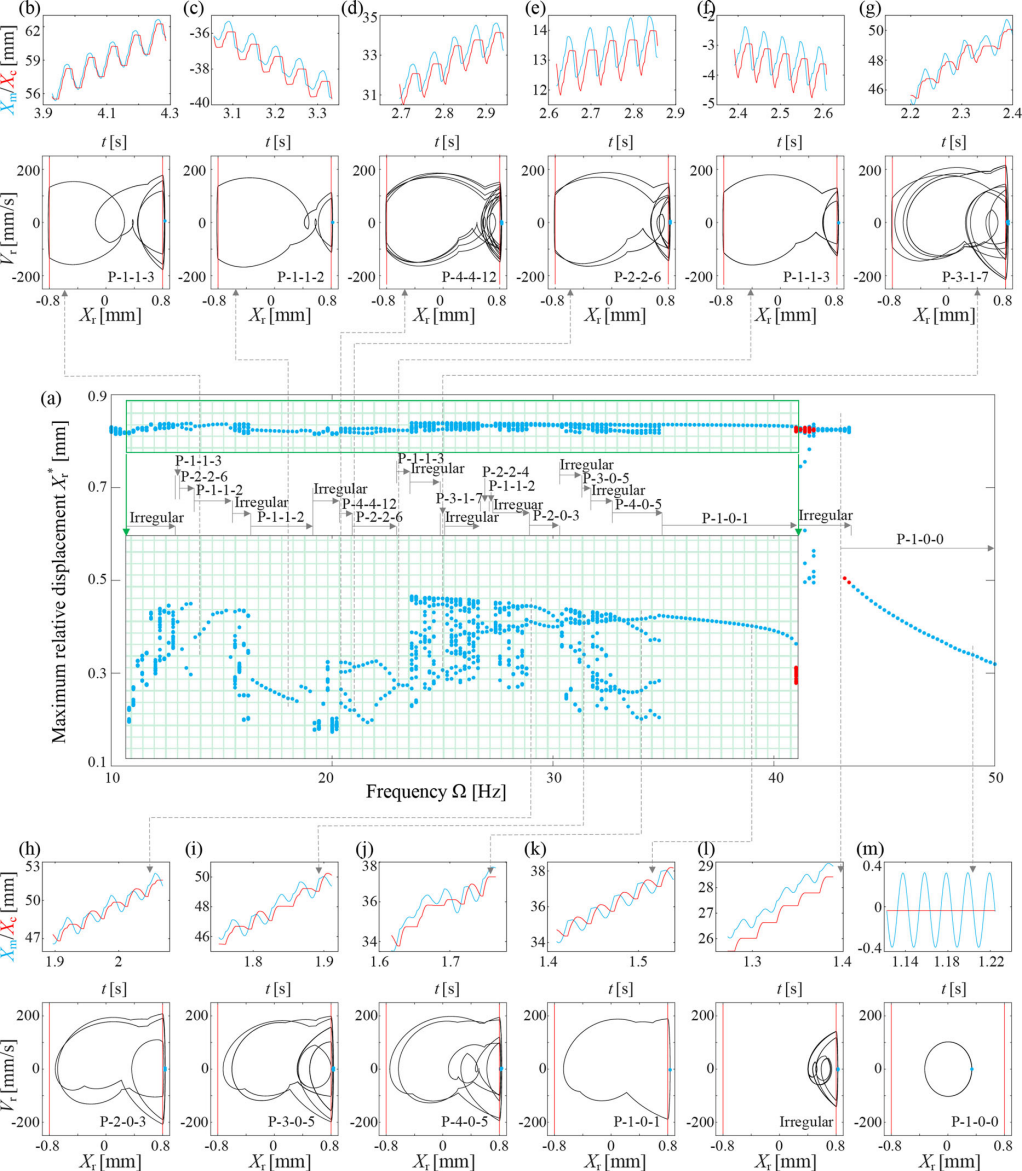
**a** Bifurcation diagram of the capsule response for $k_1=0.9$ kN/m, where the maximum relative displacement, $X_\textrm{r}$, is displayed as a function of the driven frequency. In addition, the region for $\Omega \in [10.8, 43.2]$ Hz has been enlarged to display the details in the green area. Corresponding time series and phase portraits for **b** $\Omega =14$ Hz, **c** 18 Hz, **d** 20.4 Hz, **e** 21 Hz, **f** 23 Hz, **g** 25 Hz, **h** 29 Hz, **i** 31.4 Hz, **j** 34 Hz, **k** 39 Hz, **l** 43.2 Hz, and **m** 49 Hz, have been added to show different capsule responses

**Fig. 6:**
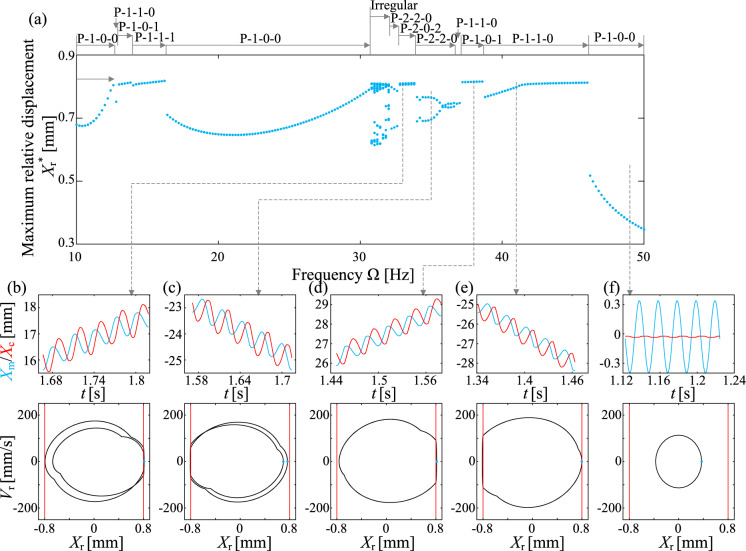
**a** Bifurcation diagram of the capsule response for $k_1=10^8$ kN/m, where the maximum relative displacement, $X_\textrm{r}$, is displayed as a function of the driven frequency. Corresponding time series and phase portraits for **b** $\Omega =33$ Hz, **c** 35 Hz, **d** 38 Hz, **e** 41 Hz, **f** 49 Hz, have been added to show different capsule responses

To unveil the intricate change in capsule speed discussed above, bifurcation diagrams corresponding to Fig. 3g–i are presented in Figs. 4, 5, and 6, respectively. In case of $X_\textrm{r}^*>G_1=0.8$ mm, the inner mass engages with the front impact spring in corresponding excitation period. To illustrate the intricacies of periodic impacts between the inner mass and the front spring, the regions for $\Omega \in [10.8, 41]$ Hz in Fig. 4 and $\Omega \in [10.8, 43.2]$ Hz in Fig. 5 have been enlarged and presented in green areas. It addition, sub-panels have been incorporated to depict time series and phase portraits of representative capsule motions, where P-*l*-*m*-*n* denotes a Period-*l* response with *m* left and *n* right impacts.

The bifurcation diagram in Fig. 4a depicts the capsule’s response for $k_1=0.3$ kN/m and $P_\textrm{d}=40$ mN, corresponding to the velocities in Fig. 3g. For low excitation frequencies, $\Omega \le 13.2$ Hz, the capsule exhibits irregular motion involving both of front and back impacts, as indicated by the time series and phase portrait shown in Fig. 4b. Around $\Omega \le 14$ Hz, the capsule transitions into a P-1-1-3 pattern, where it impacts the front spring three times per period. Simultaneously, another P-1-1-2 motion emerges, coexisting with the original P-1-1-3 motion. This coexisting P-1-1-2 motion undergoes period doubling at $\Omega \in [14.8,15.2]$ Hz, resulting in P-2-2-4 pattern, which eventually reverts back to P-1-1-2. For $\Omega \in [16,16.4]$ Hz, there co-exists another irregular motion, and the P-1-1-2 response bifurcates into P-1-1-1 for $\Omega \ge 16.4$ Hz. It then changes in to P-2-2-3 for $\Omega \le 19.6$ Hz, except the irregular motion for $\Omega =18$ Hz and P-2-4-6 motion for $\Omega \le 18.2$ Hz, and the co-existing irregular motions for $\Omega \in [18.4,19.6]$ Hz. For $\Omega \in [19.8,26.4]$ Hz, the capsule motion varies between irregular and various periodic motions, including P-2-2-8, P-1-1-4, P-2-2-6, P-1-1-3, and P-2-2-3 responses, and none of them has fast progression. Then P-1-0-1 motion arises for $\Omega \in [24.4,35.6]$ Hz, which corresponds to the branch with the fastest progression speed in Fig. 3g. A typical time series and phase portrait of this motion for $\Omega =26$ Hz is presented in Fig. 4j. As the driven frequency increases further, the capsule response transitions into P-2-1-2 and irregular motions, accompanied by a decrease in progression speed. Finally, for high-frequency excitation, $\Omega \ge 48.4$ Hz, the capsule exhibits P-1-0-0 motion, where it experiences no impact with either the front or the back springs, effectively rendering it immobile.

Compared to Figs. 4 and  5 for $k_1=0.3$ kN/m and $P_\textrm{d}=40$ mN, which features a hysteresis loop in the *von Mises* truss, unveils a richer tapestry of irregular motions. These irregular motions interlace with various periodic patterns, including P-1-1-3, P-2-2-6, P-1-1-2, P-4-4-12, P-3-1-7, P-2-2-4, P-2-0-3, P-3-0-5, P-4-0-5 responses for $\Omega \le 34.8$ Hz. Notably, P-1-0-1 motion arises for $\Omega \ge 50$ Hz, for relatively fast forward capsule progression depicted in Fig. 3. This high-speed progression gradually diminishes with increasing driven frequency. Additionally, a unique irregular response characterised by solely right impacts emerges for $\Omega \in [41, 43.4]$ Hz. This peculiar behavior exhibits a progression speed comparable to the P-1-0-1 responses. As the phase portrait displayed in Fig. 5l, this irregular response maintains positive relative displacement, i.e., $X_\textrm{r}>0$, indicating that the inner mass refrains from crossing the hysteresis loop, preventing transitions to the other branch of the *von Mises* truss. Finally, the capsule halts and exhibits a P-1-0-0 response for high-frequency excitation.

Next, the bifurcation diagram for $k_1=10^8$ kN/m and $P_\textrm{d}=40$ mN, which corresponds to the capsule velocities in Fig. 3, is presented in Fig. 6, revealing a considerably simpler pattern compared to the previous two cases. A plenty of period-one motions, involving either left or right impacts, emerge for $\Omega <30.6$ Hz. However, these motions exhibit negligible capsule progression. Except the region for $\Omega \in [30.8,32]$ Hz where the response is irregular, the capsule progression speed steadily increases for $\Omega \le 41$ Hz, driven by P-2-2-0, P-2-0-2, P-1-1-0, and P-1-0-1 patterns. The progression direction is determined by the impact spring. Among these, the fastest forward progression is achieved by P-1-0-1 for $\Omega \in [37.2,38.6]$ Hz, consistent with the preceding scenarios. For high-frequency excitation, the capsule with no impact remains immobile.

**Fig. 7:**
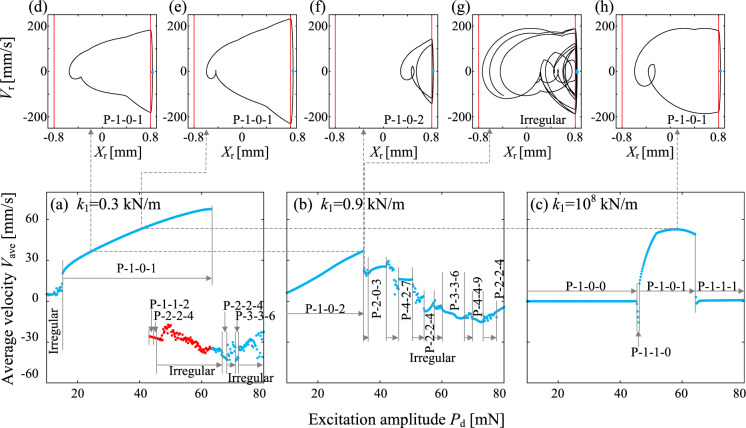
Bifurcation diagrams of the capsule response for **a** $k_1=0.3$ kN/m, **b** 0.9 kN/m and **c** $10^8$ kN/m, where the maximum relative displacement, $X_\textrm{r}$, is displayed as a function of the driven amplitude. Panels **d** $k_1=0.3$ kN/m and $P_\textrm{d}=24.6$ mN, **e** $k_1=0.3$ kN/m and $P_\textrm{d}=40.2$ mN, **f** $k_1=0.9$ kN/m and $P_\textrm{d}=34.6$ mN, **g** $k_1=0.3$ kN/m and $P_\textrm{d}=34.8$ mN, and **h** $k_1=10^8$ kN/m and $P_\textrm{d}=57.8$ mN

Based on the preceding discussion, it seems that the key to achieving the fastest forward progression lies in establishing the P-1-0-1 motion. To further explore this behaviour, we consider the driven amplitude as a control parameter and investigate the modulation of this motion. With the driven frequency fixed as $\Omega =29$ Hz, we calculated the average capsule velocities corresponding to the proceeding three *von Mises* trusses. As shown in Fig. 7a, c, the case of $k_1=0.3$ kN/m for the spring with negative stiffness exhibits the most extensive region, $P_\textrm{d}\in [15.2,63]$ mN, where P-1-0-1 motion prevails. In contrast, the linear spring supports the P-1-0-1 motion for higher-amplitude excitation, $P_\textrm{d}\in [46.2,64.2]$ mN. For the linear case in Fig. 7c, the peak velocity, $V_\text {ave}=52.76$, is achieved by $P_\textrm{d}=57.8$ mN, with a corresponding phase portrait is displayed in Fig. 7h. Interestingly, Fig. 7e reveals that the same velocity can be attained by $P_\textrm{d}=40.2$ mN in case of negative stiffness, requiring a significantly reduced driven force compared with the classical vibro-impact capsule. By contrast, the case with hysteresis loop does not yield the P-1-0-1 response in Fig. 7b. Instead, its fast progression relies on the P-1-0-2 motion for low driven amplitude, $P_\textrm{d}\le 34.6$ mN. This motion closely resembles the irregular motion pattern in Fig. 5l, which does not cross the hysteresis loop and vibrate exclusively on the right branch of the *von Mises* spring, $X_\textrm{r}>0$. Further increasing the excitation leads to large-amplitude vibrations, where the inner mass repeatedly traverses the hysteresis loop. This results in a diverse array of periodic motions interspersed with irregular responses, accompanied by a decline in progression speed as the driven amplitude escalates.

## Numerical investigation of the capsule system using path-following techniques

**Fig. 8:**
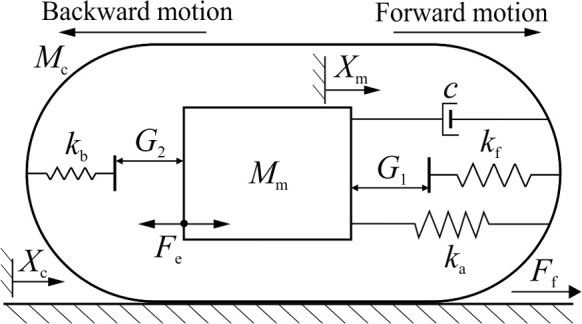
Physical model of the vibro-impact capsule system with linear structural spring studied in [16]

In this section we will carry out a numerical study of the periodic response of both the capsule model with the nonlinear *von Mises* spring described earlier and the classical capsule robot using a linear structural spring, see Fig. 8. For this purpose, we will employ the numerical continuation platform COCO [49], which allows the implementation of path-following algorithms for piecewise-smooth dynamical systems. In this investigation, one of the main concerns will be to compare the performance of the different capsule configurations in order to determine optimal operation regimes considering energy consumption and achievable average capsule velocities.

### Nondimensionalisation and numerical approximation of the nonlinear *von Mises* truss

Before we begin with the numerical investigation of the capsule model Eq. (3), we introduce the following nondimensional parameters and variables,

8 $$\begin{aligned} \Omega _{0}= &   \sqrt{\dfrac{k_{2}}{M_\textrm{m}}}, \quad \tau = \Omega _{0}t, \quad \omega = \dfrac{2\pi \Omega }{\Omega _{0}}, \nonumber \\ \xi= &   \dfrac{c}{2M_\textrm{m}\Omega _{0}}, \quad A= \dfrac{P_\textrm{d}}{P_\textrm{f}},\nonumber \\ x_{\text {m}}= &   \dfrac{k_{2}}{P_\textrm{f}}X_\textrm{m}, \quad x_{\text {c}}= \dfrac{k_{2}}{P_\textrm{f}}X_\textrm{c}, \quad x_{\text {r}}= \dfrac{k_{2}}{P_\textrm{f}}X_\textrm{r}, \nonumber \\ g_{1}= &   \dfrac{k_{2}}{P_\textrm{f}}G_{1}, g_{2}= \dfrac{k_{2}}{P_\textrm{f}}G_{2},\nonumber \\ \widetilde{L}_{0}= &   \dfrac{k_{2}}{P_\textrm{f}}L_{0}, \quad \widetilde{H}_{0}= \dfrac{k_{2}}{P_\textrm{f}}H_{0}, \quad \widetilde{k}_\text {f}= \dfrac{k_\text {f}}{k_{2}}, \nonumber \\ \widetilde{k}_\text {b}= &   \dfrac{k_\text {b}}{k_{2}}, \quad \widetilde{k}_{1}= \dfrac{k_{1}}{k_{2}},\nonumber \\ \gamma= &   \dfrac{M_\textrm{m}}{M_\textrm{c}}. \end{aligned}$$

In the present work, the vectors $z=(v_{\text {m}},x_{\text {r}},v_{\text {r}})^T\in \mathbbm {R}^3$ and $\lambda =(\omega ,A,\gamma ,k_\text {f},k_\text {b},k_{1},\xi ,g_{1},g_{2},L_{0},H_{0})\in \times \mathbbm {R}^{11}$ will denote the phase variables and parameters of the system, respectively. Here, we have omitted the tildes to simplify notation. Although the capsule model will be formulated using nondimensional parameters and variables, the numerical results will be presented in dimensional form so as to better understand the practical implications.

**Fig. 9:**
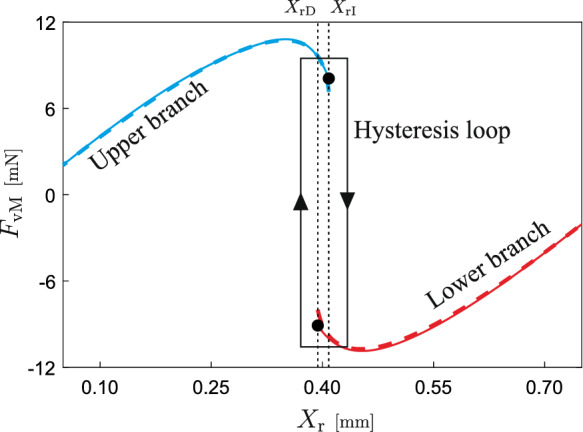
Restoring force provided by the nonlinear *von Mises* truss. The dashed line represents the force obtained by solving numerically the *von Mises* system Eq. (5), while the solid line gives the force calculated from the interpolation function Eqs. (9)–(10). The narrow rectangle stands for the hysteresis cycle observed around the interval $[X_\textrm{rD},X_\textrm{rI}]$

It is important to note that for a thorough examination of capsule dynamics, the numerical solution of the *von Mises* system (5) is crucial at each time step. However, this direct numerical approach can be computationally burdensome, particularly when employed in path-following simulations. To circumvent this computational challenge, we propose an alternative strategy that utilises a pre-computed force profile. Given that the parameters governing the physical properties of the nonlinear *von Mises* truss remain constant throughout our numerical investigation, we can solve the defining system once for a sufficiently extensive range of the position variable. This provides us with a comprehensive map of the force-displacement relationship. We then approximate this force profile using a nonlinear model that can be evaluated efficiently, enabling us to simulate capsule dynamics without the need for repeated numerical integration of the *von Mises* system:

9 $$\begin{aligned} F_\text {vM}(x_{\text {r}})=(1-H_{\text {vM}})F_\text {vM-U}(x_{\text {r}})+H_{\text {vM}}F_\text {vM-L}(x_{\text {r}}), \end{aligned}$$

where

10 $$\begin{aligned} \begin{aligned} F_\text {vM-U}(x_{\text {r}})&=\beta _{0}x_{\text {r}}e^{\eta _{0}x_{\text {r}}}+\beta _{1}x^2_{\text {r}} e^{\eta _{1}x_{\text {r}}}\\&\quad +\beta _{2}x^3_{\text {r}}e^{\eta _{2}x_{\text {r}}}+ \beta _{3}x^5_{\text {r}}e^{\eta _{3}x_{\text {r}}},\\ F_\text {vM-L}(x_{\text {r}})&=\alpha _{0}(x_{\text {r}}-2H_{0})e^{d_{0}(x_{\text {r}}-2H_{0})}\\&\quad +\alpha _{1} (x_{\text {r}}-2H_{0})^2e^{d_{1}(x_{\text {r}}-2H_{0})}\\&\quad +\alpha _{2}(x_{\text {r}}-2H_{0})^3 e^{d_{2}(x_{\text {r}}-2H_{0})}\\&\quad +\alpha _{3}(x_{\text {r}}-2H_{0})^5e^{d_{3}(x_{\text {r}}-2H_{0})}. \end{aligned} \end{aligned}$$

In this model, the functions $F_\text {vM-U}$ and $F_\text {vM-L}$ represent the upper and lower branches of the force profile given in Fig. 9. Equation (9) includes a discrete variable $H_{\text {vM}}$ that can take the values 0 or 1, depending on whether the *von Mises* truss operates on the upper or lower branches, respectively, according to the rule described below.

Suppose that the nonlinear spring is initially operating on the upper branch $F_\text {vM-U}$, i.e. $H_{\text {vM}}=0$. During this regime, the relative mass position $x_{\text {r}}$ is monitored until a critical value $X_\textrm{rI}$ is crossed from below with nonzero velocity. After this event, the *von Mises* truss experiences a sudden transition to the lower branch $F_\text {vM-L}$, and $H_{\text {vM}}$ becomes 1. Along this operation mode, the variable $x_{\text {r}}$ is once more monitored until another critical value $X_\textrm{rD}$ ($X_\textrm{rD}<X_\textrm{rI}$, see Fig. 9) is crossed from above with nonzero velocity. At this point, the *von Mises* truss again suffers an instantaneous transition, this time to the upper branch ($H_{\text {vM}}=0$), and the process repeats itself. Notice that within the interval $[X_\textrm{rD},X_\textrm{rI}]$ the upper and lower branches overlap each other, hence giving rise to a hysteresis loop as depicted in Fig. 9. Therefore, if the system initiates with an $x_{\text {r}}$ within this interval, there is ambiguity as to the *von Mises* truss operation mode (upper or lower branch). In this work, we adopt the convention that in such case the *von Mises* truss will start its operation on the upper branch given by the function $F_\text {vM-U}$, corresponding to $H_{\text {vM}}=0$. If, on the contrary, at the beginning one has that $x_{\text {r}}<X_\textrm{rD}$ or $x_{\text {r}}>X_\textrm{rI}$, no ambiguity occurs, and the values $H_{\text {vM}}=0$ and $H_{\text {vM}}=1$ are assigned, respectively.

In the remainder of this section, unless otherwise specified, we will employ the following parameters for the nonlinear *von Mises* truss model given by Eqs. (9)–(10) will be used: $\beta _{0}=0.677$, $\eta _{0}=-0.01237$, $\beta _{1}=-0.038$, $\eta _{1}=0.1644$, $\beta _{2}=-0.01698$, $\eta _{2}=4.098$, $\beta _{3}=0.07933$, $\eta _{3}=2.458$, $\alpha _{0}=0.6702$, $d_{0}=0.01262$, $\alpha _{1}=0.04013$, $d_{1}=-0.162$, $\alpha _{2}=-0.01174$, $d_{2}=-3.647$, $\alpha _{3}=0.04633$, $d_{3}=-2.078$, $X_\textrm{rI}=1.2614$ and $X_\textrm{rD}=1.2157$. Note that these parameters are given in nondimensional units. To restore the physical dimensions, Eq. (9) requires re-scaling using the following transformations: $F_\text {vM}\leftarrow F_\text {vM}P_\textrm{f}$, $x_{\text {r}}\leftarrow \dfrac{P_\textrm{f}}{k_{2}}x_{\text {r}}$, $X_\textrm{rI}\leftarrow \dfrac{P_\textrm{f}}{k_{2}}X_\textrm{rI}$ and $X_\textrm{rD}\leftarrow \dfrac{P_\textrm{f}}{k_{2}}X_\textrm{rD}$. The resulting force profiles, after re-scaling, are illustrated in Fig. 9.

### Mathematical formulation for path-following analysis

In order to study the capsule system using numerical continuation with COCO, the model has to be formulated in the framework of piecewise-smooth dynamical systems. For this purpose, the equations of motion of the capsule robot can be written in compact form as follows:

11 $$\begin{aligned} z'&=\left( \begin{array}{cc} A\sin (\omega \tau )-f_{0}-H_{\mathrm {k_\text {f}}}f_{1}-H_{\mathrm {k_\text {b}}}f_{2}& \qquad A\sin (\omega \tau )-f_{0}{-}H_{\mathrm {k_\text {f}}}f_{1}{-}H_{\mathrm {k_\text {b}}}f_{2} \\   & \qquad - \gamma \vert {H_{\textrm{vel}}}\vert \left( f_{0}{+}H_{\mathrm {k_\text {f}}}f_{1}{+}H_{\mathrm {k_\text {b}}}f_{2}{-}H_{\textrm{vel}}\right) \\ v_{\text {r}}\\ \end{array}\right) \nonumber \\&=f_{\tiny {\text {CAP}}}(z,\lambda ,H_{\mathrm {k_\text {f}}},H_{\mathrm {k_\text {b}}},H_{\text {vM}},H_{\textrm{vel}}), \end{aligned}$$

where parameters and variables are given in nondimensional form following (8). Here, the derivative with respect to the nondimensional time $\tau$ is represented by the prime symbol, and we define $f_{0}=F_\text {vM}(x_{\text {r}})+2{\xi }v_{\text {r}}$, $f_{1}=k_\text {f}(x_{\text {r}}-g_{1})$, $f_{2}=k_\text {b}(x_{\text {r}}+g_{2})$, with $F_\text {vM}(x_{\text {r}})$ being the nondimensional *von Mises* truss introduced in (9). In addition, the capsule model Eq. (11) includes the flags $H_{\mathrm {k_\text {f}}}$, $H_{\mathrm {k_\text {b}}}$ and $H_{\textrm{vel}}$, which stand for discrete variables defining the operation modes of the system, according to the rules

12 $$\begin{aligned} H_{\mathrm {k_\text {f}}}= &   {\left\{ \begin{array}{ll} 1, &  x_{\text {r}}-g_{1}\ge 0,~~\text{(contact } \text{ with }~k_\text {f}\text{) },\\ 0, &  x_{\text {r}}-g_{1}<0,~~\text{(no } \text{ contact) }, \end{array}\right. } \end{aligned}$$

13 $$\begin{aligned} H_{\mathrm {k_\text {b}}}= &   {\left\{ \begin{array}{ll} 1, &  x_{\text {r}}+g_{2}\le 0,~~\text{(contact } \text{ with }~k_\text {b}\text{) },\\ 0, &  x_{\text {r}}+g_{2}>0,~~\text{(no } \text{ contact) }, \end{array}\right. } \end{aligned}$$

14 $$\begin{aligned} H_{\textrm{vel}}= &   {\left\{ \begin{array}{ll} 0, &  v_{\text {c}}=0~ \text{ and } ~\vert {f_{0}+H_{\mathrm {k_\text {f}}}f_{1}+H_{\mathrm {k_\text {b}}}f_{2}}\vert \le 1,\\ & \text{(capsule } \text{ stationary) },\\ 1, &  v_{\text {c}}>0~ \text{ or }~\left( v_{\text {c}}=0~ \text{ and } ~f_{0}+H_{\mathrm {k_\text {f}}}f_{1}\right. \\ &  \left. \quad +H_{\mathrm {k_\text {b}}}f_{2}>1\right) ,\\ & \text{(forward } \text{ motion) },\\ -1, &  v_{\text {c}}<0~ \text{ or }~\left( v_{\text {c}}=0~ \text{ and } ~f_{0}+H_{\mathrm {k_\text {f}}}f_{1}\right. \\ &  \left. \quad +H_{\mathrm {k_\text {b}}}f_{2}<-1\right) ,\\ & \text{(backward } \text{ motion) }, \end{array}\right. } \end{aligned}$$

where $v_{\text {c}}=v_{\text {m}}-v_{\text {r}}$ gives the absolute capsule velocity. In the formulation introduced above, the term $f_{\textrm{mc}}=f_{0}+H_{\mathrm {k_\text {f}}}f_{1}+H_{\mathrm {k_\text {b}}}f_{2}$ stands for the force exerted on the capsule by the inner mass. According to Eq. (14), the capsule leaves its resting state provided the force term $f_{\textrm{mc}}$ crosses the boundary $f_{\textrm{mc}}=1$ (from below) or $f_{\textrm{mc}}=-1$ (from above), in such a way that the capsule starts moving forward or backward, respectively.

**Table 2:** Operation modes of the capsule system and the corresponding values of the discrete variables $H_{\mathrm {k_\text {f}}}$, $H_{\mathrm {k_\text {b}}}$, $H_{\text {vM}}$ and $H_{\textrm{vel}}$ defined in Eqs. (9) and (12)–(14)

Operation mode	$H_{\mathrm {k_\text {f}}}$	$H_{\mathrm {k_\text {b}}}$	$H_{\text {vM}}$	$H_{\textrm{vel}}$
$I_{1}=\left\{ \text{ NCk },\text{ Vc0 },F_\text {vM-U}\right\}$	0	0	0	0
$I_{2}=\left\{ \text{ NCk },\text{ Vcp },F_\text {vM-U}\right\}$	0	0	0	1
$I_{3}=\left\{ \text{ NCk },\text{ Vcn },F_\text {vM-U}\right\}$	0	0	0	-1
$I_{4}=\left\{ \text{ NCk },\text{ Vc0 },F_\text {vM-L}\right\}$	0	0	1	0
$I_{5}=\left\{ \text{ NCk },\text{ Vcp },F_\text {vM-L}\right\}$	0	0	1	1
$I_{6}=\left\{ \text{ NCk },\text{ Vcn },F_\text {vM-L}\right\}$	0	0	1	-1
$I_{7}=\left\{ \text{ Ck2 },\text{ Vc0 },F_\text {vM-U}\right\}$	0	1	0	0
$I_{8}=\left\{ \text{ Ck2 },\text{ Vcp },F_\text {vM-U}\right\}$	0	1	0	1
$I_{9}=\left\{ \text{ Ck2 },\text{ Vcn },F_\text {vM-U}\right\}$	0	1	0	-1
$I_{10}=\left\{ \text{ Ck2 },\text{ Vc0 },F_\text {vM-L}\right\}$	0	1	1	0
$I_{11}=\left\{ \text{ Ck2 },\text{ Vcp },F_\text {vM-L}\right\}$	0	1	1	1
$I_{12}=\left\{ \text{ Ck2 },\text{ Vcn },F_\text {vM-L}\right\}$	0	1	1	-1
$I_{13}=\left\{ \text{ Ck1 },\text{ Vc0 },F_\text {vM-U}\right\}$	1	0	0	0
$I_{14}=\left\{ \text{ Ck1 },\text{ Vcp },F_\text {vM-U}\right\}$	1	0	0	1
$I_{15}=\left\{ \text{ Ck1 },\text{ Vcn },F_\text {vM-U}\right\}$	1	0	0	-1
$I_{16}=\left\{ \text{ Ck1 },\text{ Vc0 },F_\text {vM-L}\right\}$	1	0	1	0
$I_{17}=\left\{ \text{ Ck1 },\text{ Vcp },F_\text {vM-L}\right\}$	1	0	1	1
$I_{18}=\left\{ \text{ Ck1 },\text{ Vcn },F_\text {vM-L}\right\}$	1	0	1	-1

In the formulation given in Eqs. (9) and (12)–(14), the discrete variables $H_{\mathrm {k_\text {f}}}$, $H_{\mathrm {k_\text {b}}}$, $H_{\text {vM}}$ and $H_{\textrm{vel}}$ are used to distinguish the operation modes of the capsule system in the numerical implementation. More specifically, each operation mode will be associated to a triple $\left\{ \Sigma ,\Delta ,\Theta \right\}$ with $\Sigma \in \left\{ \text{ NCk },\text{ Ck1 },\text{ Ck2 }\right\}$ (no contact with springs, contact with $k_\text {f}$, contact with $k_\text {b}$), $\Delta \in \left\{ \text{ Vc0 },\text{ Vcp },\text{ Vcn }\right\}$ (no capsule motion, forward motion, backward motion) and $\Theta \in \left\{ F_\text {vM-U},F_\text {vM-L}\right\}$ (upper branch of the *von Mises* truss force, lower branch of the *von Mises* truss force). For example, the operation mode $\left\{ \text{ Ck1 },\text{ Vcn },F_\text {vM-L}\right\}$ indicates that the capsule moves backwards with the inner mass making contact with the spring $k_\text {f}$ and the restoring force provided by the *von Mises* springs given by its lower branch (see Fig. 9). In this way, the capsule motion is characterised by 18 different operation modes, as introduced in Table 2.

Finally, for the study of the capsule model via path-following methods, it is convenient to write the external harmonic excitation in autonomous form. To this end, we append to the equations of motion a well-known nonlinear oscillator given by [52]:

15 $$\begin{aligned} {\left\{ \begin{array}{ll} r'(t)=r(t)+\omega s(t)-r(t)\left( r(t)^2+s(t)^2\right) ,\\ s'(t)=s(t)-\omega r(t)-s(t)\left( r(t)^2+s(t)^2\right) , \end{array}\right. } \end{aligned}$$

which possesses the asymptotically stable solution $r(t)=\sin (\omega t)$, $s(t)=\cos (\omega t)$, for all $t\ge 0$. In this way, we can write the whole system in autonomous form, which then allows us to study the model via numerical continuation methods in a straightforward manner.

### Solution measures

In the subsequent section, we will study the behaviour of the capsule model under variations of excitation frequency and amplitude. To effectively analyze the robot’s performance and identify optimal operating regimes, it is beneficial to introduce suitable solution measures. To this end, we define the (nondimensional) average velocity per period ($T=2\pi /\omega$) of the capsule as follows:

16 $$\begin{aligned} V_{\tiny {\text {AVG}}}=\frac{1}{T}(x_{\text {c}}(T)-x_{\text {c}}(0)). \end{aligned}$$

According to Eq. (8), the dimensional average velocity can be obtained by applying the transformation $V_{\tiny {\text {AVG}}}\leftarrow V_{\tiny {\text {AVG}}}P_\textrm{f}\Omega _{0}/k_{2}$. The sign of the average velocity indicates the direction of capsule movement: positive for forward motion and negative for backward motion. Note that system(11) does not explicitly represent the capsule motion $x_{\text {c}}$. However, this variable can be retrieved from Eq. (11) using the following relationship:

$$\begin{aligned} x_{\text {c}}(\tau )  &   =x^*_{\text {c}}+\int \limits _{0}^{\tau }v_{\text {c}}(s)\,ds \\  &   = x^*_{\text {c}}+\int \limits _{0}^{\tau }(v_{\text {m}}(s) - v_{\text {r}}(s))\,ds, \end{aligned}$$

where $x^*_{\textrm{c}}\in \mathbbm {R}$ represents the position of the capsule at $\tau =0$. The second physical measure that will be considered in our study is the average power used to drive the internal mass $M_\textrm{m}$ per period, computed (without dimensions) as

17 $$\begin{aligned} P_{\tiny {\text {AVG}}}=\frac{1}{T}\int \limits _{0}^{T}A\sin (\omega s)v_{\text {m}}(s)\,ds, \end{aligned}$$

where $P_{\tiny {\text {AVG}}}\leftarrow P_{\tiny {\text {AVG}}}P^2_\textrm{f}\Omega _{0}/k_{2}$ should be replaced in order to obtain its value with units of power. The introduced solution measures Eqs. (16) and (17) provide valuable insights into the dynamics of the capsule and enable a more practical understanding of its behavior. As will be demonstrated in the following section, these measures will aid in identifying optimal operating regimes and assessing the robot’s performance under varying conditions.

**Fig. 10:**
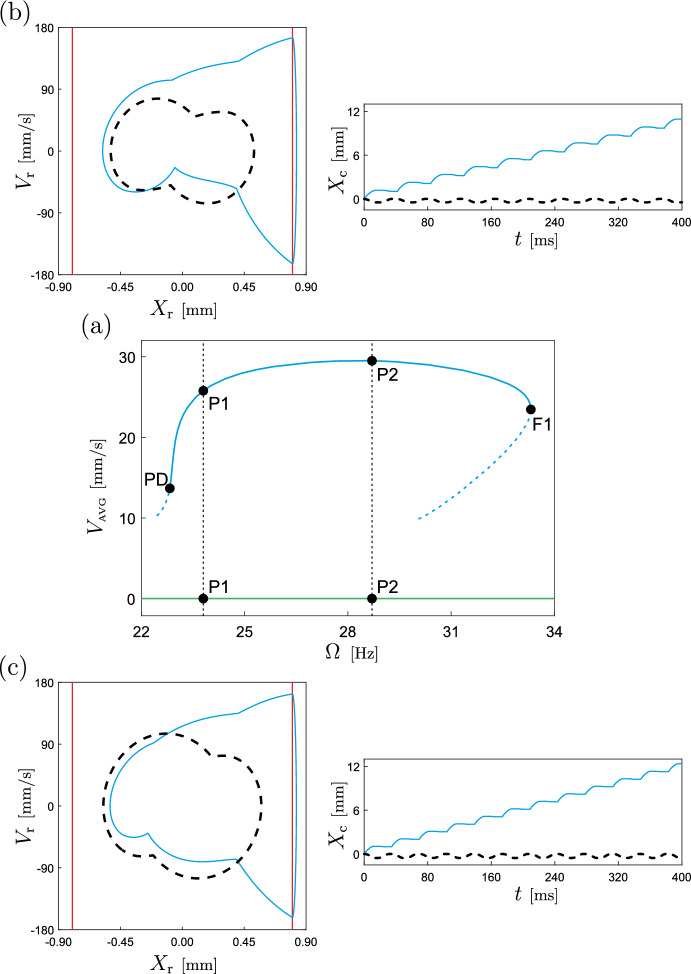
**a** Numerical continuation with respect to the excitation frequency of periodic orbits of the capsule system (11) (considering the nonlinear *von Mises* truss, in blue) and of the classical capsule model with linear springs (in green), computed for the parameter values given in Table 1. The vertical axis shows the average capsule velocity during one excitation period. The labels PD and F1 mark period-doubling and fold bifurcations of limit cycles, located at $\Omega \approx 22.8268$ Hz and $\Omega \approx 33.3149$ Hz, respectively. In this diagram, solid and dashed lines represent stable and unstable periodic solutions, respectively. Panels **b**, **c** display solutions of the capsule system (11) (solid, blue) and the classical capsule model (dashed, black), calculated at the test points P1 ($\Omega =23.8$ Hz) and P2 ($\Omega =28.7$ Hz), respectively. In this diagram, the solutions of the capsule model with the *von Mises* truss posses the solution signature $\left\{ I_{5},I_{2},I_{1},I_{3},I_{1},I_{4},I_{16},I_{17}\right\}$, as defined in Table 2. In the phase plots, the vertical red lines represent the impact boundaries $x_{\text {r}}=g_{1}$ and $x_{\text {r}}=-g_{2}$

### Numerical study

In this section we will carry out a numerical investigation of the capsule response based on the mathematical formulation introduced in Sect. 4.2 (see Eq. (11)), using the numerical continuation platform COCO [49]. Specifically, we utilise the continuation approach to compare the performance of two capsule configurations: the one introduced in the previous section, featuring the nonlinear *von Mises* truss, and the classical capsule configuration employing a linear structural spring, as depicted in Fig. 8. To ensure a fair comparison, we employ identical parameter sets for both configurations. Having these considerations in mind, we begin our numerical study with the continuation of the period-1 response of the capsule with respect to the excitation frequency. The results are presented in Fig. 10. This diagram depicts the path-following of periodic solutions for both capsule configurations, with blue representing the capsule incorporating the nonlinear *von Mises* spring (Eq. (11)) and green representing the classical configuration. The figure demonstrates the behaviour of the capsule’s average velocity per excitation period as a function of the frequency. As can be observed, for the chosen parameter values, only system Eq. (11) is capable of propelling the capsule forward, achieving a maximum capsule velocity of approximately 29.51 mm/s at $\Omega \approx 28.1334$ Hz. The continuation process reveals the existence of two codimension-1 bifurcations of limit cycles: period-doubling (PD, $\Omega \approx 22.8268$ Hz) and fold (F1, $\Omega \approx 33.3149$ Hz). These critical points therefore define a window of stability for the observed period-1 response with nonzero forward motion. In contrast, the classical capsule model proves ineffective in perturbing the main capsule body from its resting position, resulting in a zero average velocity ($V_{\tiny {\text {AVG}}}=0$) across the entire frequency range under investigation.

**Fig. 11:**
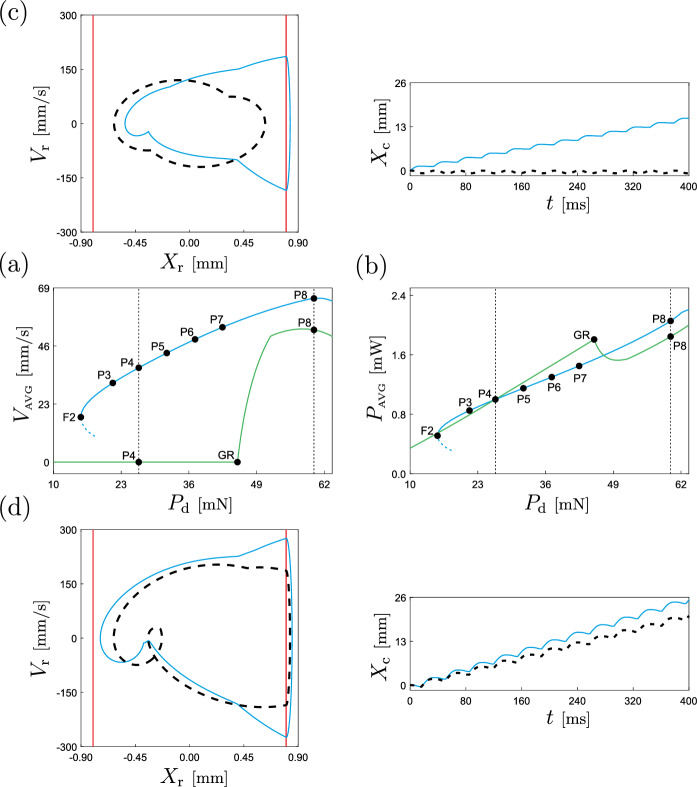
**a** Numerical continuation with respect to the excitation amplitude $P_\textrm{d}$ of periodic orbits of the capsule system (11) (considering the nonlinear *von Mises* truss, in blue) and of the classical capsule model with linear springs (in green), computed for the parameter values given in Table 1. The vertical axis shows the average capsule velocity during one excitation period. The labels F2 and GR mark fold and grazing bifurcations of limit cycles, located at $P_\textrm{d}\approx 15.2589$ mN and $P_\textrm{d}\approx 45.3237$ mN, respectively. In this diagram, solid and dashed lines represent stable and unstable periodic solutions, respectively. **b** Continuation outcome of panel **a** showing on the vertical axis the average power consumption during one excitation period. The test points P3–P7 represent the amplitude values for which the power consumption equals 0.85 mW ($P_\textrm{d}\approx 21.3936$ mN), 1.00 mW ($P_\textrm{d}\approx 26.3727$ mN), 1.15 mW ($P_\textrm{d}\approx 31.7516$ mN), 1.30 mW ($P_\textrm{d}\approx 37.1813$ mN) and 1.45 mW ($P_\textrm{d}\approx 42.4451$ mN), respectively. Panels **c** and **d** display solutions of the capsule system (11) (solid, blue) and the classical capsule model (dashed, black), calculated at the test points P4 and P8 ($P_\textrm{d}=60$ mN), respectively

Next, we fix the excitation frequency and study the evolution of the periodic response with respect to the amplitude of external excitation $P_\textrm{d}$. The findings are presented in Fig. 11. Panel (a) depicts the behaviour of the average capsule velocity as $P_\textrm{d}$ varies, utilizing blue and green hues to differentiate solutions obtained with the *von Mises* capsule system (11) and the classical capsule model, respectively. For the considered range, system (11) exhibits an increasing trend of capsule velocity with rising $P_\textrm{d}$, reaching a peak value of $V_{\tiny {\text {AVG}}}\approx 64.93$ [mm/s] for $P_\textrm{d}\approx 61.0117$ mN. By contrast, the classic capsule model remains stationary for over half the considered amplitude spectrum, only propelling the capsule forward upon encountering a grazing bifurcation of limit cycles GR ($P_\textrm{d}\approx 45.3237$ [mN]). This critical point corresponds to a periodic solution grazing the spring $k_\text {f}$ (i.e. $x_\textrm{r}-g_{1}=0$ with $v_\textrm{r}=0$). Thereafter, the capsule velocity increases with the excitation amplitude, but consistently remains below the velocity levels attained by system (11). Figure 11b shows the behaviour of the average power dissipated over one excitation period $P_{\tiny {\text {AVG}}}$ for both capsule configurations. As evident from this diagram, the power required to operate the capsule in both cases is essentially identical, with the main disparity being the inferior performance of the classical capsule configuration in terms of capsule movement.

**Fig. 12:**
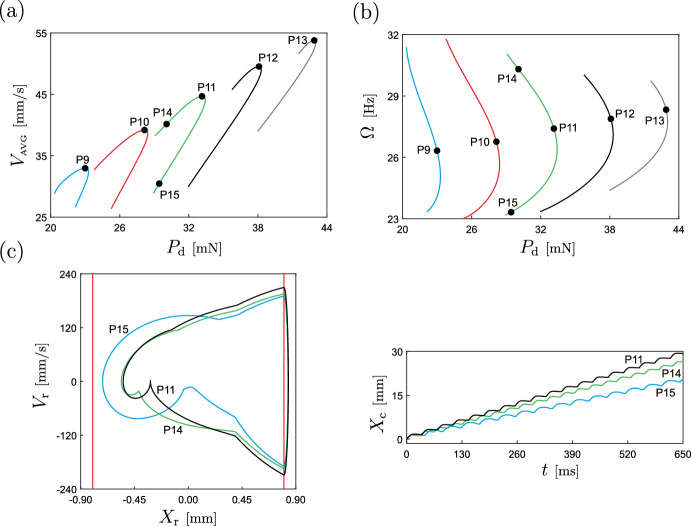
Two-parameter continuation with respect to frequency and amplitude of excitation of the periodic solutions calculated at the points P3 (blue curve), P4 (red curve), P5 (green curve), P6 (black curve) and P7 (grey curve) shown in Fig. 11b, maintaining a constant average power consumption. Panel **a** shows the behaviour of the average capsule velocity $V_{\tiny {\text {AVG}}}$, while panel **b** presents the computed curves on the amplitude-frequency plane. The points P9 ($P_\textrm{d}\approx 22.9921$ mN, $\Omega \approx 26.3265$ Hz), P10 ($P_\textrm{d}\approx 28.1484$ mN, $\Omega \approx 26.7656$ Hz), P11 ($P_\textrm{d}\approx 33.1426$ mN, $\Omega \approx 27.4077$ Hz), P12 ($P_\textrm{d}\approx 38.0934$ mN, $\Omega \approx 27.8833$ Hz), and P13 ($P_\textrm{d}\approx 42.8955$ mN, $\Omega \approx 28.3309$ Hz) are points at which the average capsule velocity is maximized, for the corresponding fixed power consumption. Panel **c** depicts system responses calculated at the points P11 (in black), P14 ($P_\textrm{d}=30.06$ mN, $\Omega =30.31$ Hz, in green) and P15 ($P_\textrm{d}=29.43$ mN, $\Omega =23.32$ Hz, in blue)

The test points P3–P7 depicted in Fig. 11b, correspond to the amplitude values for which the average power consumption for the capsule model with the *von Mises* truss is 0.85 mW ($P_\textrm{d}\approx 21.3936$ mN), 1.00 mW ($P_\textrm{d}\approx 26.3727$ mN), 1.15 mW ($P_\textrm{d}\approx 31.7516$ mN), 1.30 mW ($P_\textrm{d}\approx 37.1813$ mN) and 1.45 mW ($P_\textrm{d}\approx 42.4451$ mN), respectively. These points will be used to perform a two-parameter continuation analysis in frequency and amplitude of excitation to identify optimal operation regimes for a given fixed energy supply. Specifically, using the continuation capabilities of COCO, we will find a locus on the amplitude-frequency plane that maintain a constant value of $P_{\tiny {\text {AVG}}}$, corresponding to the specified values. Subsequently, we will monitor the behaviour of the average capsule velocity $V_{\tiny {\text {AVG}}}$ along these loci in search of the maximum value. The results of this procedure are presented in Fig. 12. Panel (b) presents the amplitude-frequency curves along which the power consumption remains constant, while panel (a) shows the corresponding capsule velocity responses. Hence, our numerical investigation reveals that for the considered fixed power levels, it is indeed possible to find suitable values of frequency and amplitude of excitation to achieve optimal capsule velocity, as demonstrated by the test case depicted in panel (c).

## Conclusions

This paper studied a novel vibro-impact capsule robot that employs a *von Mises* truss, replacing the conventional linear spring, to improve robot’s progression speed. After a concise discussion on the stiffness property of the *von Mises* truss, a new capsule robot model was proposed to study its dynamics. Bifurcation analysis revealed that the fastest forward progression was achieved by the period-1 motion featuring no left impact and a single right impact. Subsequently, a path-following method was introduced to track this motion and optimise the robot’s progression speed.

The *von Mises* truss connects one linear spring with two inclined springs. According to the combination of the spring stiffness and geometric properties, the *von Mises* can exhibit properties of negative stiffness, hysteresis loop, or approximately linear stiffness. The degenerated case, the *von Mises* with an approximately linear stiffness, can be regarded as a representation of the classical vibro-impact capsule studied in our previous works, e.g., [6, 19].

Our bifurcation analysis demonstrated that neither extremely high nor low excitation frequencies yield rapid progression. Low-frequency excitation often induces non-periodic impacts, resulting in a relatively low progression speed. High-frequency excitation produces periodic motions, but the absence of impacts leads to the robot oscillating at its original location. For the capsule designed in this study, parameter sweeping found its highest-speed progression realised by the driven frequency around 30 Hz. For negative and approximately linear stiffness scenarios, the fastest progression was realised by a period-1 response with no left impact and one right impact. Additionally, the negative stiffness configuration achieved the same speed with a smaller excitation amplitude. In contrast, the case with a hysteresis loop exhibited more intricate responses, particularly when the excitation was strong enough for the *von Mises* truss to cross the hysteresis loop. However, these motions involving hysteresis loop crossings proved inefficient. The fastest progression in this case was achieved by a small-amplitude periodic motion with right spring impact only, devoid of snap-backs.

Further analysis of the period-1 motion was conducted using a path-following continuation method implemented in COCO. This analysis corroborated the benefits of incorporating the *von Mises* truss, as it enabled the same progression speed as the classical vibro-impact capsule robot when the driving force was significantly reduced. Finally, a two-parameter continuation was performed on the frequency-amplitude plane, demonstrating the maximum robot speed achievable for a specified power consumption.

The present work is our preliminary study of the new vibro-impact capsule robot in combination with the *von Mises* truss. Our future work will concentrate on three key areas: firstly, theoretical research involving the substitution of front and rear constraint plates with *von Mises truss* spring systems [53], aiming to elucidate the influence of nonlinearity on the propelling response. Secondly, we aim to develop continuum nonlinear structures as replacements for *von Mises* trusses, encompassing their fabrication and seamless integration with the capsule robot prototype. Lastly, we will embed active materials within these continuum nonlinear structures, rendering them reprogrammable to attain *physical intelligence* [54].

## Data Availability

The datasets generated and analysed during the current study are not publicly available due to the potential use for further publications, but are available from the corresponding author on reasonable request.

## List of symbols

$$\mu $$: Frictional coefficient (–)
$\Omega$: Frequency of the driven force (Hz)
*c*: Damping between the innear mass and the capsule (Ns/m)
$u_\text {apx}$: Apex displacement of the *von Mises* truss (m)
$F_\text {e}$: External driven force (mN)
$F_\text {v}$: Reaction force of the *von Mises* truss (mN)
$G_1$: Front gap between the innear mass and the capsule (mm)
$G_2$: Back gap between the innear mass and the capsule (mm)
$H_0$: Rise of the *von Mises* truss (mm)
$k_1$: Stiffness of inclined linear spring in the *von Mises* truss (kN/m)
$k_2$: Stiffness of third linear spring in the *von Mises* truss (kN/m)
$k_b$: Stiffness of the back impact spring (kN/m)
$k_f$: Stiffness of the front impact spring (kN/m)
$L_0$: Span of the *von Mises* truss (mm)
$M_\text {m}$: Mass of the capsule shell (g)
$M_\text {m}$: Mass of the inner mass (g)
$P_\text {d}$: Magnitude of the driven force (mN)
$V_\text {r}$: Relative velocity between the inner mass and the capsule (mm)
$X_\text {r}$: Relative displacement between the inner mass and the capsule (mm)
$X_\text {r}^*$: Maximum relative displacement in each driven period (mm)
$V_\text {ave}$: Average capsule velocity (mm/s)
